# Longitudinal Association Between Self-Reported Chewing Difficulty and Depression Among Older Japanese Adults Using Data From the Longevity Improvement and Fair Evidence Study

**DOI:** 10.7759/cureus.84753

**Published:** 2025-05-24

**Authors:** Takashi Miyano, Ryosuke Kaneko, Akihiro Kishimura, Takahisa Anada, Masako Hosoi, Michiko Furuta, Koichiro Kato, Haruhisa Fukuda

**Affiliations:** 1 Department of Medical and Robotic Engineering Design, Tokyo University of Science, Tokyo, JPN; 2 Department of International and Community Oral Health, Tohoku University Graduate School of Dentistry, Sendai, JPN; 3 Department of Bioscience and Biotechnology, Faculty of Agriculture, Kyushu University, Fukuoka, JPN; 4 Department of Applied Chemistry, Faculty of Engineering, Kyushu University, Fukuoka, JPN; 5 Center for Future Chemistry, Kyushu University, Fukuoka, JPN; 6 Center for Molecular Systems, Kyushu University, Fukuoka, JPN; 7 Department of Chemistry and Biochemistry, Kyushu University, Fukuoka, JPN; 8 Institute for Materials Chemistry and Engineering, Kyushu University, Fukuoka, JPN; 9 Department of Psychosomatic Medicine, Kyushu University Hospital, Fukuoka, JPN; 10 Section of Preventive and Public Health Dentistry, Division of Oral Health, Growth and Development, Faculty of Dental Science, Kyushu University, Fukuoka, JPN; 11 Department of Health Care Administration and Management, Kyushu University, Fukuoka, JPN

**Keywords:** aged adult, chewing ability, claims data, depression, longitudinal studies, oral health

## Abstract

Introduction

Depression in older adults is linked to adverse health outcomes such as reduced daily living activities and decreased quality of life. Although studies show a connection between depression and oral health issues like chewing difficulty, the specific links between subjective chewing ability, the number of teeth, and the onset of depression remain underexplored. This study aimed to investigate the relationship between self-reported chewing ability and the onset of depression among an elderly population aged 65 years or older in Japan.

Methods

Using claim data from Japan's National Health Insurance, we conducted a longitudinal study of 23,417 subjects aged 65 years and older. These subjects participated in specific health checkups and visited a dental office within one year (April 2018 to March 2019). Participants who reported, "Sometimes it is difficult to chew due to dental problems" or "I can hardly chew" were identified as having chewing difficulties. The number of teeth was estimated using dental care claims data. We employed multivariate logistic regression analyses, with new diagnoses of depression from April 2019 to March 2020 as the dependent variable and self-reported chewing difficulty and the number of teeth as independent variables.

Results

Out of the total 23,417 participants, 462 (2.0%) received a new diagnosis of depression during the one-year follow-up period. After adjusting for covariates, self-reported chewing difficulty was significantly associated with the onset of depression (odds ratio = 1.26; 95% confidence interval: 1.00-1.58). However, there was no significant association between the number of teeth and depression.

Conclusion

Among individuals aged 65 years and older, self-reported chewing difficulty has a significant impact on the risk of developing depression. This study underscores the importance of addressing oral health, particularly chewing ability, to mitigate the risk of depression.

## Introduction

In Japan, the population is aging rapidly, with projections indicating that one in three individuals will be over 65 years old by 2030 [1]. Depression, a major public health concern, is one of the most prevalent diseases globally and is associated with significant economic burdens [2]. In older adults, depression is linked to several adverse health outcomes, including diminished daily living activities, reduced quality of life, and increased risk of suicide [3].

Research consistently demonstrates a connection between depression and oral health issues in older adults, particularly chewing difficulty [4]. Various mechanisms may underpin this relationship, such as alterations in neurotransmitter balance, eating patterns, and social activity [5]. Cross-sectional studies in Japan have identified both subjective and objective chewing problems as factors associated with depression [6]. A longitudinal study suggests that subjective chewing issues may contribute to the development or exacerbation of depressive symptoms in individuals aged 65 and older [7]. Chewing ability can decline due to aging and various oral conditions, including tooth loss, the number and pattern of occlusal contacts (the contact between opposing teeth in the upper and lower jaws), occlusal force, and decreased saliva secretion [8]. While previous studies have noted a correlation between chewing difficulties and depression, the specific links between chewing ability, number of teeth, and the onset of depression have not been thoroughly documented.

It is crucial to investigate the modifiable factors associated with depression, as this knowledge can aid medical professionals in preventing and managing the condition. The current study aimed to examine whether self-reported chewing ability and the number of teeth are linked to new diagnoses of depression among an elderly population aged 65 years or older in Japan.

## Materials and methods

Study design and data sources

This investigation utilized data from the Longevity Improvement & Fair Evidence (LIFE) study [9]. The LIFE study, started in 2019, is a longitudinal cohort database project that systematically collects and correlates administrative claims and Specific Health Checkup data from residents in participating municipalities. The analytical framework encompasses three main components: (i) National Health Insurance claims data, (ii) claims data from the latter-stage healthcare system for older persons, and (iii) Specific Health Checkup data.

Study population

We accessed anonymized claims data from four LIFE Study municipalities in Japan that provided consistent data throughout the study period.

**Figure 1:**
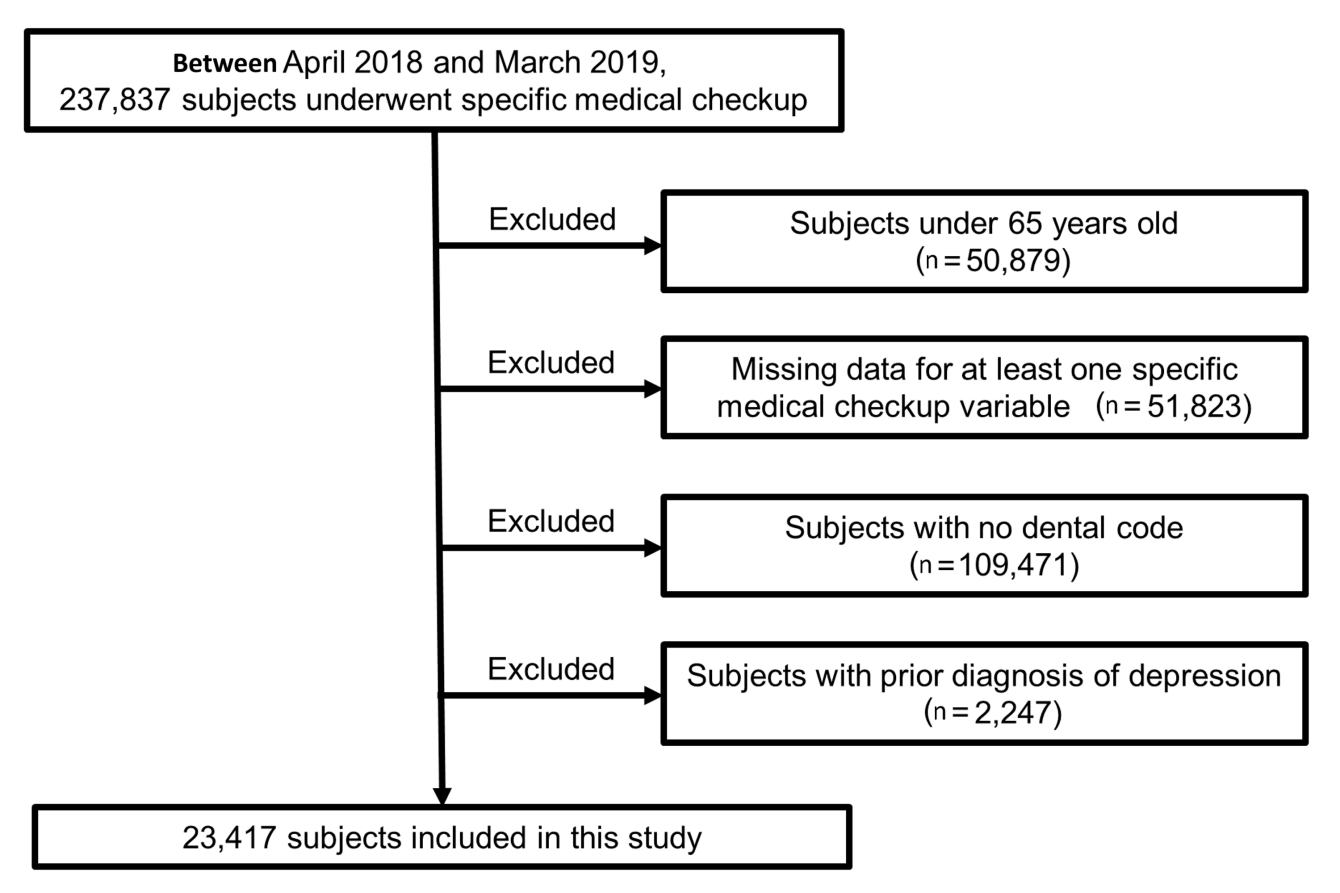
Flow diagram of sample selection This flowchart outlines the process of selecting participants from the LIFE Study claims database [9]. The initial pool included 237,837 individuals with available health checkup data. Exclusion criteria were applied in the following order: participants under 65 years of age (n=50,823), those with missing health checkup data (n=51,823), those lacking dental codes for 28 teeth within one year before the health checkup data (n=109,471), and individuals diagnosed with depression within 12 months preceding the index diagnosis (n=2,247).

Figure 1 shows a flowchart of the participant selection method. Initially, individuals who underwent Specific Health Checkups between April 2018 and March 2019 were identified, totaling 237,837 participants. From this group, we excluded individuals under the age of 65 (n=50,879), those with incomplete data for at least one Specific Health Checkup variable, such as date of birth, date of medical examination, sex, body mass index (BMI), Charlson comorbidity index (CCI), smoking status, alcohol consumption, and sleep (n=51,823), and those lacking a dental code for a complete set of 28 teeth (n=109,471). Subjects previously diagnosed with depression within the last year were also excluded (n=2,247). The identification of depression diagnoses was based on the International Classification of Diseases, 10th Revision (ICD-10) codes: F32 (major depressive disorder, single episode) and F33 (major depressive disorder, recurrent), as detailed in a previous report [10]. Ultimately, 23,417 participants were included in the analysis.

Outcome

The outcome was newly diagnosed depression. Newly diagnosed cases were identified using the following criteria: (i) candidates were categorized based on a documented diagnosis of F32 or F33 in their medical claims data throughout the follow-up period (from the date of the Specific Health Checkup to March 2020), and (ii) those with a suspected F32 or F33 diagnosis and deaths during the follow-up period were excluded.

Explanatory variables

Chewing problems were assessed as a self-reported symptom using the Specific Health Checkup questionnaire, which asked, "Which of the following statements most accurately describes your ability to eat or chew food?". Participants who selected "Sometimes it is difficult to chew due to dental problems" and "I can hardly chew" were considered to have chewing difficulties [11], the latter category being small in number.

While the LIFE Study dataset did not include specific information on the number of teeth, patients who visited dental care facilities and had dental codes for all teeth recorded in the context of management, encompassing periodontal examinations or mechanical tooth cleaning covered by insurance during the investigation period, were included in the analysis. If more than two dental codes for each tooth were registered between April 2018 and March 2019, the most recent results were used for analytical purposes. The number of teeth present was computed using a six-digit dental code that provided information on tooth position and condition, specifically the presence or absence of teeth. Previous research has validated the accuracy of tooth count recorded in claims data by cross-referencing it with data from a national survey conducted in Japan [12]. The number of teeth, excluding third molars, was estimated and categorized into three groups (20 or more, 10-19, or < 10).

Other variates

In a meta-analysis of community-dwelling elderly individuals, several factors associated with the risk of depression were identified, including experiences such as the loss of a loved one, disruptions in sleep patterns, physical limitations, previous depressive episodes, and being of the female sex [13]. We controlled for age, sex (male or female), BMI, comorbid disease history, smoking, alcohol consumption, and sleep, which may correlate with depression and could be extracted from the LIFE Study dataset. Age was calculated as the difference between the date of birth and the date of the medical checkup. Since age was not normally distributed, as determined by the Shapiro-Wilk test, subjects were stratified into four age groups: 65-69, 70-74, 75-79, and ≥ 80 years. BMI is defined as participant weight in kilograms divided by the square of participant height in meters and is often used as a parameter of nutritional status. A BMI < 18.5 kg/m^2^ is accepted as underweight, a BMI of 18.5-24.9 kg/m^2^ as normal weight, and a BMI ≥ 25.0 kg/m^2^ as overweight. We assessed comorbid disease history using the CCI. Participants were divided into three groups based on their CCI scores: 0, 1, and ≥ 2. Smoking status was classified according to current smoking status. Current smokers were defined as those who had smoked more than five packs (100 cigarettes) in their lifetime and had smoked daily or occasionally in the past 28 days. Alcohol consumption was classified based on frequency, with current drinkers defined as those who drank alcohol daily or occasionally. Sleep status was classified based on self-reported restfulness from sleep. Participants were asked the question, “Do you feel well-rested after sleeping?” If a participant answered “yes,” they were categorized as having good sleep status. Conversely, participants who answered “no” were classified as having poor sleep status.

Statistical analysis

In the descriptive analysis, continuous variables were presented as means with corresponding standard deviations, and categorical variables were presented as counts and percentages. The percentages of new depression onset in five-year age groups, by sex, BMI, medication history, smoking, and alcohol consumption, were calculated. Comparisons of depression percentages across these factors were performed using chi-squared tests. Logistic regression analysis was employed to calculate the adjusted odds ratios (ORs) and the corresponding 95% confidence intervals (95% CIs) for depression. In the first model, chewing ability and number of teeth were adjusted for sex and age. In the second model, chewing ability and number of teeth were entered simultaneously and adjusted for age, sex, and sleep, all of which were significantly associated with depression, as shown in Table 1. In the third model, the covariates included age, sex, BMI, CCI, smoking, alcohol, and sleep. R Studio, version 4.0.4 (R Foundation for Statistical Computing, Vienna, Austria), was used for all analyses, with statistical significance set at p < 0.05.

**Table 1:** Characteristics in patients with and without depression ^†^P for chi-square test

Variables	Non-Depression (n=22,955)			Depression (n=462)			^†^P-value
	Frequency	Percentage		Frequency		Percentage	
Age group							
65–69 years	8,528		98.4%	140	1.6%		0.004
70–74 years	10,292		98.0%	215	2.0%		
75–79 years	2,325		97.6%	57	2.4%		
80– years	1,810		97.3%	50	2.7%		
Sex							
Male	9,040		98.2%	163	1.8%		0.074
Female	13,915		97.9%	299	2.1%		
BMI							
18.5–24.9	2,013		97.8%	45	2.2%		0.73
< 18.5	16,338		98.0%	328	2.0%		
≥ 25.0	4,604		98.1%	89	1.9%		
Charlson comorbidity index							
0	20,207		98.0%	421	2.0%		0.12
1	1,710		98.4%	27	1.6%		
≥ 2	1,038		98.7%	14	1.3%		
Smoking							
No	21,319		98.0%	433	2.0%		0.48
Yes	1,636		98.3%	29	1.7%		
Alcohol							
No	5,426		98.3%	96	1.7%		0.15
Yes	17,529		98.0%	366	2.0%		
Sleep							
Good	17,422		98.1%	329	1.9%		0.020
Poor	5,533		97.7%	133	2.3%		
Chewing ability							
Good	18,424		98.1%	350	1.9%		0.016
Poor	4,531		97.6%	112	2.4%		
Number of teeth							
≥ 20	18,364		98.1%	363	1.9%		0.75
10–19	2,242		97.9%	48	2.1%		
< 10	2,349		97.9%	51	2.1%		

## Results

A total of 23,417 subjects were included in the analysis (9,203 male and 14,214 female) with a mean age of 71.5 ± 4.8 years. Of this, 462 (2.0%) patients (163 male and 299 female) with a mean age of 72.3 ± 5.2 years were newly diagnosed with depression.

The relationship between dental status and five-year age groups is detailed in Table 2. The number of teeth decreased with age; participants older than 80 years had an average of 19.7 ± 9.7 teeth. The percentage of participants reporting chewing difficulty was 17.4% in the 65-69 age group, 19.9% in the 70-74 age group, 20.4% in the 75-79 age group, and 30.0% in the ≥80 years age group, showing an age-dependent increase.

**Table 2:** Oral condition in different age groups Data are given as mean±SD for continuous variables and frequency (percentages) for categorical variables

Variables	Total (N = 23,417)	65–69 years (n=8,668)	70–74 years (n=10,507)	75–79 years (n=2,382)	≥80 years (n=1,860)
Age (years), mean±SD	71.5±4.8	67.3±1.4	71.7±1.4	76.3±1.4	83.6±3.4
Sex, n (%)					
Male	9,203 (39.3%)	3,133 (36.1%)	4,326 (41.2%)	951 (39.9%)	793 (42.6%)
female	14,214 (60.7%)	5,535 (63.9%)	6,181 (58.8%)	1,431 (60.1%)	1,067 (57.4%)
Number of teeth, n (%)					
≥ 20	18,727 (80.0%)	7,476 (86.3%)	8,360 (79.6%)	1,783 (74.8%)	1,108 (59.6%)
10–19	2,290 (9.8%)	619 (7.1%)	1,065 (10.1%)	302 (12.7%)	304 (16.3%)
< 10	2,400 (10.2%)	573 (6.6%)	1,082 (10.3%)	297 (12.5%)	448 (24.1%)
Number of teeth, mean±SD	23.8±7.6	25.0±6.5	23.7±7.7	22.8±8.1	19.7±9.7
Chewing ability, n (%)					
Good	18,774 (80.2%)	7,161 (82.6%)	8,416 (80.1%)	1,895 (79.6%)	1,302 (70.0%)
Poor	4,643 (19.8%)	1507 (17.4%)	2091 (19.9%)	487 (20.4%)	558 (30.0%)

The characteristics related to chewing difficulty are presented in Table 3. A total of 18,774 (80.2%) had good abilities and 4,643 (19.8%) had poor chewing abilities. Those with chewing difficulty had higher rates of depression (p = 0.019). Each covariate, except for the CCI, was significantly affected by chewing ability (p < 0.001).

**Table 3:** Distribution of participant characteristics according to chewing difficulties ^†^P for chi-square test

Variables	Good chewing ability (n=18,774)			Poor chewing ability (n=4,643)			^†^P-value
	Frequency	Percentage		Frequency		Percentage	
Depression							
No	18,424		98.1%	4,531	97.6%		0.019
Yes	350		1.9%	112	2.4%		
Age groups (years)							
65–69	7,161		38.1%	1,507	32.5%		< 0.001
70–74	8,416		44.8%	2,091	45.0%		
75–79	1,895		10.1%	487	10.5%		
≥80	1,302		6.9%	558	12.0%		
Sex							
Male	7,113		37.9%	2,090	45.0%		< 0.001
Female	11,661		62.1%	2,553	55.0%		
BMI (kg/m^2^)							
18.5–24.9	13,475		71.8%	3,191	68.7%		< 0.001
< 18.5	1,583		8.4%	475	10.2%		
≥ 25.0	3,716		19.8%	977	21.0%		
Charlson comorbidity index							
0	16,507		87.9%	4,121	88.8%		0.21
1	1,420		7.6%	317	6.8%		
≥ 2	847		4.5%	205	4.4%		
Smoking							
No	17,686		94.2%	4,066	87.6%		< 0.001
Yes	1,088		5.8%	577	12.4%		
Alcohol							
No	4,299		23.6%	1,223	20.8%		< 0.001
Yes	14,475		76.4%	3,420	79.2%		
Sleep							
Good	14,654		78.1%	3,097	66.7%		< 0.001
Poor	4,120		21.9%	1,546	33.3%		
Number of teeth							
≥ 20	16,040		85.4%	2,687	57.9%		< 0.001
10–19	1,316		7.0%	974	21.0%		
< 10	1,418		7.6%	982	21.2%		

The results of the logistic regression analysis for depression are shown in Table 4. Participants with poor chewing ability (reference: good chewing ability) had OR of 1.26 (95%CI 1.00-1.58), with a p-value of 0.046. Participants with 11-19 teeth had OR 0.96 (95%CI 0.70-1.31) and those with fewer than 10 teeth had OR 0.97 (95%CI 0.71-1.32), with p-values of 0.79 and 0.83, respectively.

**Table 4:** Multivariate logistic regression analysis for depression Model 1: Chewing ability and number of teeth were adjusted for sex and age group; Model 2: Chewing ability and the number of teeth were entered simultaneously and adjusted for age, sex, and sleep. OR, odds ratio; CI, confidence interval

Variables	Model 1			Model 2		
	OR	95% CI	P-value	OR	95% CI	P-value
Chewing ability (reference: Good)						
Poor	1.28	1.03–1.59	0.025	1.26	1.00–1.58	0.046
Number of teeth (reference: ≥ 20)						
10–19	1.02	0.76–1.39	0.879	0.96	0.70–1.31	0.79
< 10	1.03	0.76–1.38	0.867	0.97	0.71–1.32	0.83

## Discussion

To the best of our knowledge, this study is the first to propose a potential association between self-reported chewing difficulty (using the Specific Health Checkup questionnaire) and an increased likelihood of depression onset among older Japanese adults. These findings align with previous research, which indicates that elderly individuals experiencing subjective chewing problems are at a higher risk of depression [7].

Subjects with self-reported chewing difficulties tended to be older than those with good chewing status (Table 2), corroborating numerous studies that have found chewing ability declines with age [14,15]. Additionally, the observed relationship between poor chewing ability and a smaller number of teeth (Table 2) validates the reliability of our results and the utility of our research methodology [14,15]. According to recent research on the association between chewing status and oral health, chewing ability correlates with several factors, including whether or not dental treatment has been received, the number of teeth present, periodontal pocket depth of 4 mm or greater, decayed teeth, and missing teeth [16]. Considering that the use of dentures affects chewing ability [17], the presence or absence of dentures may significantly impact these subjects and should be factored into assessments of total functional tooth units. Another possibility is that patients have become accustomed to a decreased number of teeth and reduced occlusal support area, and thus do not experience discomfort.

The effect of subjective chewing problems on depression was observed after adjusting for the number of teeth (Table 4). However, the mechanisms underlying the relationship between chewing problems and depression remain poorly understood. There are several plausible explanations for this association. First, chewing difficulty may reduce the release of neurotransmitters such as serotonin and dopamine, which are crucial for mood regulation. Generally, adequate levels of these neurotransmitters are associated with a positive mood, while imbalances are linked to conditions such as depression. Chewing and oral-buccal movements are reported to activate serotonergic neurons in a subgroup of dorsal raphe in the cat [18]. A previous study reported that prolonged rhythmic chewing increases serotonin levels, contributing to a neuronal mechanism that reduces stress perception [19]. However, a recent systematic umbrella review reported that mood disorders are unlikely to be directly caused by low serotonin levels [20]. Second, difficulties in chewing and eating can lead to changes in dietary patterns, prompting the selection of inappropriate foods and food textures depending on oral condition, potentially resulting in loss of pleasure and nutritional deficiencies [21,22]. This is particularly significant in older adults, in whom a lack of eating enjoyment could play an important role in frailty and malnutrition, leading to depression [23]. Poor nutrition and dietary patterns are linked to an increased risk of depression [24]. For instance, deficiencies in certain vitamins, such as vitamin B12, and the ratio of omega-6 to omega-3 fatty acids [25]. Third, difficulty in chewing may affect the ability to enjoy social activities, such as sharing meals with others, including relatives and friends. According to a survey of older Japanese people, chewing difficulties not only reduce social interactions, such as opportunities to eat with someone, but also increase the risk of depression [26]. In Japanese community-dwelling older adults, social isolation and a lack of enjoyable activities are recognized risk factors for depression [27].

Tooth loss is associated with depression, and becoming edentulous is linked with an increased risk of developing depressive symptoms [26,28]. In this study, no significant association was observed between depression and the number of teeth (Table 4). Compared with the Japanese National Survey of dental diseases [12], the participants in the current study maintained a relatively higher number of teeth across all age groups (Table 2), which may have underestimated the impact of the number of teeth on depression. The number of teeth alone does not fully represent oral function. A previous study suggested that subjective chewing difficulty might be a more reliable indicator than measured dental status itself [29]. Collectively, these findings suggest that self-reported chewing ability, as assessed by the Specific Health Check-up questionnaire, may be a useful parameter for investigating potential associations between dental factors and depression.

Although the primary strength of this study was its large sample size of National Health Insurance enrollees, it had several limitations. First, the cohort consisted of patients who had visited a dentist and undergone a specific medical checkup. Therefore, due to selection bias, the results of this study cannot be generalized to all older adults in Japan. Second, variations in depression severity could not be considered. The method of depression diagnosis (e.g., whether a formal diagnostic interview was used) may have varied between patients. Furthermore, because this study used a clinical diagnosis of depression and only included patients with major depression or depressive episodes, it provided a conservative estimate of the number of patients with depression. Third, the number of teeth was calculated from the dental code, and information regarding the presence or type of dentures (e.g., removable or fixed) was not obtained. Posterior teeth and their occlusal contact play an important role in chewing ability [8], and patients who have lost their posterior teeth are generally rehabilitated with removable dental prostheses and partial dentures to improve their chewing ability. The masticatory efficiency of removable dentures is generally lower than that of fixed dentures, and variations in masticatory ability related to denture type were not accounted for in this study. Additionally, in 2018, a new item, self-reported chewing ability, was introduced into the Japanese health checkup questionnaire, making data on chewing ability before this revision unavailable. Lastly, there may be residual confounding factors, as depression is often related to socioeconomic variables, such as retirement, social isolation, poor economic status, and bereavement, particularly in older people [13]. Future studies should investigate whether socioeconomic variables are associated with chewing ability and depression.

## Conclusions

Depression in older adults is a critical public health issue that not only worsens health outcomes but also drives up healthcare costs, independent of underlying medical conditions. Although this study could not definitively establish a causal link between subjective chewing difficulties and depression, it revealed a clear temporal association between self-reported chewing problems and subsequent new diagnoses of depression. This finding underscores the potential role of oral health, particularly masticatory function, in the mental well-being of the elderly. Simple, questionnaire-based assessments of chewing ability may serve as valuable tools in the early identification of individuals at risk. Dental professionals should proactively engage in evaluating and improving chewing function, which may contribute to preventive strategies against late-life depression.
